# Nanostructured Ti-13Nb-13Zr alloy for implant application—material scientific, technological, and biological aspects

**DOI:** 10.3389/fbioe.2023.1255947

**Published:** 2023-08-24

**Authors:** Lina Klinge, Lukas Kluy, Christopher Spiegel, Carsten Siemers, Peter Groche, Débora Coraça-Huber

**Affiliations:** ^1^ Institute for Materials Science, TU Braunschweig, Braunschweig, Germany; ^2^ Institute for Production Engineering and Forming Machines, TU Darmstadt, Darmstadt, Germany; ^3^ Research Laboratory for Biofilms and Implant Associated Infections (BIOFILM LAB), Experimental Orthopedics, University Hospital for Orthopedics and Traumatology, Medical University of Innsbruck, Innsbruck, Austria

**Keywords:** Ti-13Nb-13Zr, nanostructured titanium, equal channel angular swaging, biofilm, *Staphylococcus aureus*, implant material, osteoblasts

## Abstract

In dentistry, the most commonly used implant materials are CP-Titanium Grade 4 and Ti-6Al-4V ELI, possessing comparably high Young’s modulus (>100 GPa). In the present study, the second-generation titanium alloy Ti-13Nb-13Zr is investigated with respect to the production of advanced dental implant systems. This should be achieved by the fabrication of long semi-finished bars with high strength and sufficient ductility to allow the automated production of small implants at low Young’s modulus (<80 GPa) to minimize stress shielding, bone resorption, and gap formation between the bone and implant. In addition, bacterial colonization is to be reduced, and bone adhesion is to be enhanced by adjusting the microstructure. To do so, a dedicated thermo-mechanical treatment for Ti-13Nb-13Zr has been developed. This includes the adaption of equal channel angular swaging, a modern process of severe plastic deformation to continuously manufacture nanostructured materials, to Ti-13Nb-13Zr and short-time recrystallization and ageing treatments. In particular, two-pass equal channel angular swaging at a deformation temperature of 150°C and a counterpressure of 8 MPa has successfully been used to avoid shear band formation during deformation and to produce long Ti-13Nb-13Zr bars of 8 mm diameter. During recrystallization treatment at 700°C for 10 min followed by water quenching, a sub-micron-size primary α-phase in a matrix of α″-phase was developed. Subsequent ageing at 500°C for 1 h leads to martensite decomposition and, thus, to a homogeneously nanostructured microstructure of α- and β-phase with substructures smaller than 200 nm. The resulting mechanical properties, especially the ultimate tensile strength of more than 990 MPa, fulfill the requirements of ASTM F1713 at Young’s modulus of 73 GPa. Biological investigations show promising results in reducing bacterial biofilm formation and increased cell proliferation of osteoblasts compared to CP-Titanium Grade 4 and Ti-6Al-4V ELI, especially, if etched surfaces are applied.

## 1 Introduction

In dentistry, first-generation titanium alloys, such as CP-Titanium Grade 4 (CP-4), a medium strength α titanium alloy, and Ti-6Al-4V ELI (Ti-64 ELI), a high strength (α+β) titanium alloy, are used due to their well-balanced mechanical properties, a comparably low Young’s modulus, corrosion resistance, and biocompatibility (Geetha et al., 2009). The two latter characteristics can be explained by the formation of a dense oxide layer at related surfaces (Lütjering and Williams, 2007). CP-4 is normally used for the production of dental implants, whereas Ti-64 ELI alloy is applied as an abutment material. Lately, especially the use of Ti-64 ELI in implant applications has been intensively discussed as aluminum (Al) is suspected to cause several diseases such as breast cancer (Darbre et al., 2011), dementia, and Alzheimer’s disease (Mirza et al., 2017). In addition, vanadium and vanadium ions as well as its oxides are cytotoxic (Geetha et al., 2009). Due to the different mechanical properties, namely, the differences in strength of the implant and abutment, relative movements between these two components are possible so that wear and abrasion might occur, leading to oxide layer removal and the release of metal particles which, in turn, can infiltrate the bloodstream. To avoid potential health problems, the so-called second-generation titanium alloys such as Ti-13Nb-13Zr (TNZ) and Ti-15Mo have been developed (Geetha et al., 2009).

Compared to other biomedical materials like CoCr-based alloys or stainless steels, Young’s modulus of first-generation titanium alloys is significantly lower (approximately 250 GPa for CoCr-alloys, 210 GPa for steels, and 110 GPa for titanium alloys). However, in comparison to the stiffness of human bones (10–30 GPa), Young’s modulus is still relatively high (approximately 100–120 GPa) so that stress shielding can occur, which might lead to bone resorption and, hence, gap formation between the bone and implant. In addition to the risk of implant loosening, bacteria can colonize in the gaps and form biofilms to shield themselves from the environment and the immune system. As a result, inflammations and infections can occur, leading to a disease called peri-implantitis. In particular, antibiotic-resistant bacteria are responsible for a large fraction of implant revisions (Chouirfa et al., 2019).

The treatment of peri-implantitis includes grinding and polishing of related implant surfaces to remove the biofilms. However, previously applied coatings or etched surfaces as typically used in dental implantology to enhance osseointegration of related titanium implants are removed as well so that after the peri-implantitis therapy, re-osseointegration (if at all) only takes place to a lesser extent. In the worst case, additional implant loosening can occur, requiring an implant revision surgery as, at present, there is no alternative treatment. Since the bone material in the jaw is limited, such a revision can only be repeated for a limited number of times. Therefore, smaller dental implants are preferential to preserve as much of the bone mass as possible. On the other hand, smaller implant diameters increase the requirements regarding the strength to avoid implant deformation or failure (Attarilar et al., 2019).

Previous studies at ultrafine-structured titanium surfaces as carried out by Attarilar et al. (2021), Park et al. (2011), Miao et al. (2017), and Cao et al. (2018) have shown promising results to inhibit bacterial growth and enhance bone formation. Taking these results to account with respect to a potential peri-implantitis therapy, a fully nanostructured material would be favorable. Due to the different mechanical properties of the grains and phases, as well as those of the grain (or phase) boundaries, after grinding and polishing of an ultrafine-grained (UFG) material, a nanostructured (slightly rough) surface would redevelop naturally supporting bone cell (re)attachment and growth, whereas the colonization of bacteria can be reduced simultaneously. If necessary, increased roughness might be produced by etching.

In summary, advanced next-generation dental implant systems should fulfill the following criteria: 1) implant and abutment should be manufactured from one alloy only to minimize the risk of relative movements, 2) the avoidance of potentially critical alloying elements should increase the biocompatibility, 3) a high-strength material should be used to avoid implant and abutment fractures and allow the production of small implants, 4) low Young’s modulus would help to reduce stress-shielding, and 5) a UFG material should be applied to promote (re)osseointegration.

Consequently, in the present study, TNZ alloy was used. First of all, the TNZ alloy does not contain any critical elements, as so far, no negative health effects of niobium and zirconium have been reported (Zhou et al., 2012). TNZ belongs to the group of β-rich (α+β) titanium alloys and can, therefore, occur in two stable phases α and β (Lütjering and Williams, 2007). In addition, the metastable α˝-martensite can form after fast cooling, e.g., water quenching from sufficient high temperatures. The α˝-martensite has an orthorhombic lattice configuration and is, therefore, a soft and well formable phase even at lower temperatures (Brunke, 2019). In addition, its Young’s modulus is relatively low, e.g., Young’s modulus of 58 GPa at an ultimate tensile strength (UTS) of 630 MPa and an *A* of 25% is present in the soft state (Klinge et al., 2022a). TNZ was introduced by Smith and Nephews in the 1990s (Davidson and Kovacs, 1992) and is specified in ASTM F1713 as an implant material. Here, a minimal UTS of 860 MPa at a minimal elongation of fracture (*A*) of 8% are specified, the so-called heat-treated state. Information on Young’s modulus is not provided in ASTM F1713. However, bone-remodeling simulations carried out by our project partners have shown that Young’s modulus between 70 and 80 GPa is beneficial for osseointegration as, on the one hand, it is low enough to prevent stress shielding during service, but, on the other hand, is high enough to avoid overloading of the newly formed bone cells during the implant ingrowth phase. To meet the requirements of ASTM F1713 and to take the aforementioned criteria into account, the following goals have been set regarding the mechanical properties of a long bar (length of more than 1 m) made of TNZ: Young’s modulus of 70–80 GPa, an UTS of at least 950 MPa, and a minimum *A* of 8%.

To produce high strength, fully nanostructured materials, severe plastic deformation (SPD) processes have been developed and introduced. Standard SPD methods include high pressure torsion (HPT) and equal channel angular pressing (ECAP) (Attarilar et al., 2019). Although, in recent years, further process development has been performed, most SPD processes are still characterized by high friction and discontinuity. Hence, applications have been limited to a laboratory scale so far or, at least, show limitations in the production of long semi-finished products. To overcome these problems and to allow the production of long bar-shaped workpieces, a research group at the Institute for Production Engineering and Forming Machines (PtU) of TU Darmstadt developed the equal channel angular swaging (ECAS) process. In ECAS, the forming tool is divided into two oscillating dies, which significantly reduces friction and, therefore, enables a continuous production of bars at an unchanged shape and geometry of the workpiece. The latter allows the process to be repeated as often as necessary to achieve very large strains incrementally and, thus, store sufficient deformation energy to promote grain refinement. Previous studies have shown that UFG materials can be continuously produced from conventional copper, steels, FeCo alloys, and CP-titanium by the application of the ECAS process (Görtan, 2014). This process is now being adapted to the TNZ alloy.

After TNZ-ECAS, due to work hardening, the strength increases, whereas the elongation at fracture decreases. Short-time recrystallization heat treatments of these deformed samples (which have been developed in the current study as well) lead to an UFG microstructure with primary-α (α_p_) grain sizes smaller than 1 μm, which is essential to reduce bacterial colonization. Depending on the recrystallization temperature, different phase compositions can be adjusted after water quenching, namely, α+β, α+β+αʺ, and α+αʺ in case recrystallization is carried out below β-transus-temperature (β_T_–approximately 720–740°C) or a fully martensitic αʺ-structure is produced once β_T_ was exceeded during this heat treatment. However, the presence of a small α_P_-phase volume fraction is desirable to prevent grain growth and to improve the fatigue properties (Lütjering and Williams, 2007). Both, the β-phase and the αʺ-martensite, offer low values of Young’s modulus which is important to prevent stress shielding. During a subsequent ageing treatment at moderate temperatures, a secondary-α (α_s_) phase can be precipitated in the β-phase leading to a nanostructure and a higher strength. In addition, the αʺ-martensite (partially) decomposes into small α_s_+β substructures during ageing and, therefore, further refines the (already nanostructured) microstructure. Hence, with the ongoing martensite decomposition, the elongation at fracture decreases and Young’s modulus increases. Therefore, a well-adjusted heat-treatment after ECAS has to be developed to meet the requirements of ASTM F1713 and to optimize the mechanical properties.

## 2 Materials and methods

In case of CP-4 and Ti-64 ELI, standard materials for implant applications were purchased and used in the as-received (AR) condition. In addition, in case of CP-4, sandblasted surfaces have been produced to roughen the surfaces for the biological tests (sCP-4). TNZ alloy used in this study was melted and re-melted (resulting in two ingots with diameter 49 mm) at GfE Metalle and Materialien GmbH in Nuremberg, Germany. The chemical composition of all the three alloys is shown in Table 1. Afterward, the TNZ ingots were radially forged to a diameter of 15 mm at an elevated temperature in several steps with intermediate annealing at GFM GmbH in Steyr, Austria. In the second step, cold radial forging was applied at GFM to further reduce the diameter to 10 mm. At PtU, rotary swaging was performed (first heat of 10 mm bars) to achieve the final diameter of 8 mm (not possible at GFM). Due to strong work hardening after the radial forging, rotary swaging caused adiabatic shear band formation. For the biological tests, shear band free, nanostructured regions of these bars were used to produce related samples. To avoid adiabatic shear band formation during rotary swaging, a second heat of bars was heat treated prior to rotary swaging at PtU to establish a fully α˝-martensitic starting structure. This was achieved by annealing above β_T_ [750°C for approximately 30 min with subsequent water quenching (WQ)]. These bars were used for all ECAS experiments and the determination of the mechanical properties.

**TABLE 1:** Chemical composition of CP-4, Ti-64 ELI, and TNZ ingots in wt%.

	Ti	Nb	Zr	Al	V	Fe	C	H	N	O
CP-4	Balance	—	—	—	—	0.02	0.051	0.004	0.004	0.34
Ti-64 ELI	Balance	—	—	6.01	3.97	0.170	0.020	—	0.0100	0.1200
TNZ ingot 1	Balance	13.21	12.89	—	—	0.022	0.014	0.003	0.003	0.065
TNZ ingot 2	Balance	13.12	12.87	—	—	0.023	0.010	0.002	0.004	0.068

For the TNZ alloy, four different stages of the forming process (different microstructures and mechanical properties) were investigated. The first stage was taken right after rotary swaging (RS) to Ø 8 mm without further heat treatment. The second stage consisted of rotary swaged material with subsequent recrystallization annealing at 700°C for 10 min followed by WQ and ageing at 500°C for 1 h with air cooling (AC). Stage 2 rods already show a nanostructured microstructure. In addition, the mechanical properties meet the requirements of ASTM F1713 (Klinge et al., 2022b). These two stages represent a potential conventional (and cheaper) process route for TNZ, providing sufficient mechanical properties at lower stiffness and improved biocompatibility compared to CP-4 and Ti-64 ELI, whereas the material might not be fully nanostructured due to the missing ECAS step (Klinge et al., 2023). For the third stage samples, an additional two-pass ECAS step between the rotary swaging and the heat-treatment has been added. The fourth stage represents the final, optimized production route consisting of pre-heat treatment (750°C/30 min/WQ) of the Ø 10 mm bars. After the additional two-pass ECAS, a second rotary swaging step was performed to straighten the bars and to smoothen the surface to a diameter of Ø 7 mm. Finally, recrystallization and ageing were performed as described for stage 2 and 3 specimens.

The different stages of forming and heat treatment steps are summarized in Table 2. In addition, stage 4 process is shown. For each condition, microstructure investigations and hardness measurements were performed. In addition, for the final stage (stage 4), the mechanical properties were determined by tensile tests.

**TABLE 2:** Summarized forming and heat treatment steps for all tested materials.

	Pre-heat treatment	Forming step	Heat treatment
TNZ stage 1	—	RS from Ø 10 mm to Ø 8 mm	—
TNZ stage 2	—	Stage 1	700°C/10 min/WQ + 500°C/1 h/AC
TNZ stage 3	—	Stage 1 + ECAS	700°C/10 min/WQ + 500°C/1 h/AC
TNZ stage 4	750°C/30 min/WQ	Stage 3 + RS to Ø 7 mm	700°C/10 min/WQ + 500°C/1 h/AC
CP-4	—	AR	—
sCP-4	—	AR + sandblasted	—
Ti-64 ELI	—	AR	—
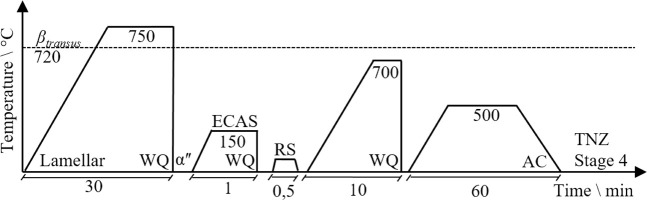			

### 2.1 Equal channel angular swaging (ECAS) process

**FIGURE 1:**
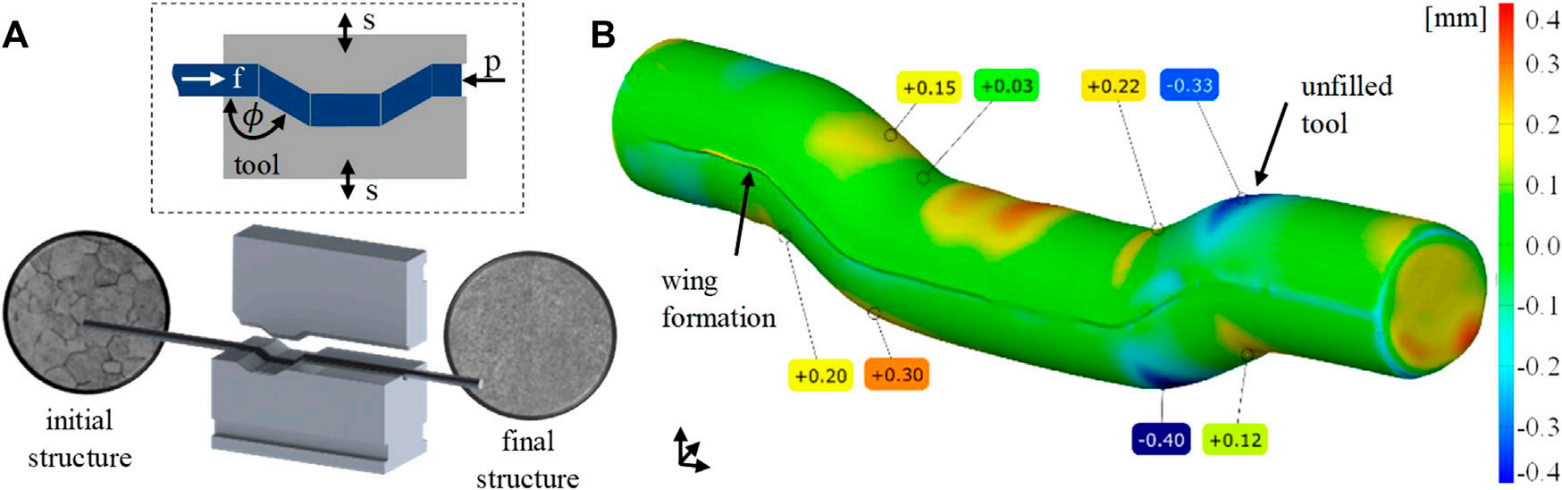
**(A)** ECAS process on a rotary swaging machine and **(B)** 3D scan of the processed bar, geometric surface comparison between reality and simulation, deviation in mm.

The ECAS process is shown in Figure 1A. Here, coarse-grained, fully martensitic TNZ bars were fed into the ECAS tools (f), which oscillate(s). During the ECAS process, the material is guided through a channel formed by the tool jaws at a dedicated feed rate. A counter pressure (p) opposite to the feed direction is applied to avoid buckling of the sheared bars. Depending on the shear angle (Φ), shear strains are introduced into the material. The output material is an ECAS-processed TNZ bar with increased strength (increase in hardness) but the same diameter as the initial bar, allowing multiple passes.

Preliminary studies at TNZ have shown that room temperature ECAS forming can lead to material defects like small surface cracks (Kluy et al., 2021). Therefore, the process was carried out at an elevated temperature. For this purpose, the forming jaws were tempered to 150°C by heating cartridges. Prior to the deformation, the TNZ bars were pre-heated to 150°C as well by means of an induction coil located directly ahead of the ECAS tools to minimize cooling during specimen progress. The feed rate was 600 mm/min at a swaging frequency of 30 Hz and a radial infeed of 0.7 mm.

Three-dimensional finite element simulations were carried out using the implicit-forming software Simufact 2021 (Figure 1B). Parameter variations were used to optimize the ECAS process with respect to a homogeneous strain distribution within the bars which, in turn, would allow the production of a fully nanostructured material. The digital sample was 70 mm long and had a diameter of 8 mm. Isotropic elastic–plastic material behavior based on the work by El-Magd and Abouridouane (2006) with temperatures of 20°C, 400°C, 600°C, and 800°C and strain rates of 0.1, 1, 10, and 100 1/s determined by cylinder compression tests was used. A coefficient of friction of 0.12 was determined in a compression slip test for the tangential, isotropic kinematic contact model between the sample and the surfaces of the tools. The tools were designed as rigid bodies without heat conduction. The counter pressure was varied between 0 MPa and 12 MPa in steps of 4 MPa.

### 2.2 Microstructure analysis and mechanical testing

For heat treatments, chamber furnaces Carbolite CWF 1,300 using standard air atmosphere were used. Rapid cooling (WQ) was achieved by taking the smaller samples and bars out of the furnace manually and dipping them into a bucket (in less than 3 s after opening the furnace) filled with approximately 10 L water for small samples and 200 L for long bars. For air cooling (AC), the samples and bars were removed from the furnace and put on building bricks.

Microstructural investigations were performed at metallographic cross-sections. The samples were sectioned by disc cutting (Jean Wirtz, Cuto 20), embedded into EpoMet^®^ amorphous, glass fiber-reinforced polymer by warm embedding (Buehler Simplimet 4,000). Afterwards, the samples were processed (ATM Saphir 550) by automated, water-cooled mechanical grinding with SiC grinding papers (size P320, P400, P600, P800, P1200, and P2500) and polishing [9 μm, 6 μm, and 3 µm with Kulzer BioDiamant^®^ diamond suspension including lubrication followed by OPS polishing with SiO_2_ OPS (25 mL) + H_2_O (10 mL) + KOH (0.7 g) + distilled water]. Finally, etching was carried out using a special titanium etching reagent (86 mL H_2_O+ 12 mL H_2_O_2_ + 4.5 mL HNO_3_ + 5 mL HF) for 3–5 s for the TNZ specimens. The CP-4 samples were etched for 20 s with the same etching reagent. The Ti-64 ELI specimens were etched with Kroll’s reagent (100 mL H_2_O+ 6 mL HNO_3_ + 3 mL HF) for 7 s.

The microstructure has been analyzed by means of scanning electron microscopy (LEO 1550) using an in-lens detector. Hardness tests (HV10) were carried out using an automated Vickers hardness tester [(LECO LV100AT), 10 indentations per specimen distributed along the diameter]. HV1 hardness tests have been performed by means of a DuraScan 2 hardness tester. The indentation time has been set to 15 s in all experiments. The measurement deviation at HV10 is ±3% and ±4% for HV1. Homogeneity testing, especially after ECAS processing, was performed by producing contour plots consisting of more than 60 data points per sample, as shown in Section 3.1. Tensile tests were carried out according to DIN EN ISO 6892-1 using a Zwick/Roell universal tensile testing machine AllroundLine (100 kN) at room temperature together with the testXpert software by Zwick. Tensile test specimens were produced according to DIN 50125 B 4 × 20. For each material condition, two specimens were tested. For a more accurate determination of the Young’s modulus a hysteresis loop was performed after 0.8% plastic strain. The pre-load was 5 MPa, the velocity for the determination of the Young’s modulus was 10 MPa/s and the testing speed was 0,006 1/s.

### 2.3 Biological testing

The samples used for biological investigations had a cylindrical geometry with a height of 6 mm and a diameter of 6 mm. All samples were prepared according to Section 2.2. Prior to use, all samples have been cleaned in sterile distilled water and were sterilized with UV-light for 1 h at 254 nm with 40 μW/cm^2^ intensity (ESCO SC2-6E1, UV-30A Lamp) to ensure similar starting conditions.

Before use, for each pre-culture of *Staphylococcus aureus ATCC 29213*, three colonies were suspended in 2 mL of TSB +1% Glu media in a 15 mL centrifuge tube (VWR International, Radnor, PA, United States). The pre-cultures were incubated in a moisture chamber at 37°C on a shaker with 200 rpm for 24 h. To ensure biofilm growth on the upper circular area of the titanium cylinders, a silicone O-ring sealed off the upper sample area fitting into a well of a 48-well plate. Six titanium samples were used for each strain. The pre-cultures were diluted 1:100 to reach an initial cell count of 10^3^ per mL media. A total of 500 μL of each culture dilution was pipetted in each well used on a shaker with 200 rpm for 24 h.

After biofilm formation, the remaining media was removed. Nine replicates for each material have been used. Following this, the titanium samples from the 48-well plate were removed and have been washed in PBS to remove planktonic bacterial cells. Then, the samples were added into sterile vials. Afterward, 500 µL of TrypLE (Thermo Fisher Scientific, Waltham, MA, United States) was added to remove bacterial cells from the titanium surface. Before and after 5 min of incubation at 37°C, each vial has been vortexed for 15 s. To inactive TrypLE, 500 µL of DMEM media has been added. Before dilution steps were initiated, each vial has been vortexed. Each vial has been diluted from 10^–1^ to 10^–8^ with TSB +1% glucose and plated 10^–6^ to 10^–8^ dilutions in triplicates on MHA plates. The freshly plated MHA plates were incubated for 48 h at 37°C. After incubation, the CFU has been counted.

Prior to osteoblast adhesion to the titanium samples used, *hFOB 1.19* cells have been pre-cultured in two passages to 90% confluency. For the first subculture, 1 × 10^4^ osteoblasts were seeded in a 75 mL cell culture flask in 10 mL of Dulbecco’s modified Eagle medium (DMEM): Nutrient Mixture F12 (Gibco, Life Technologies, United States) in combination with 10% FCS and 1% of Pen-Strep. The pre-culture got incubated at 34°C at 95% humidity and 5% of CO_2_ to 90% confluency. After 90% confluency was reached, the cells were passaged, starting with 1 × 10^4^ cells, and were then incubated up to 90% confluency. The media got changed every 48 h.

After 90% confluency was reached of the second passage, the cells were extracted for adhesion on the titanium samples used. To do so, the old media were removed, the cells have been washed in 5 mL phosphate-free PBS, and after removing the PBS, 5 mL of TrypLE has been added (Thermo Fisher Scientific, Waltham, MA, United States). After 5 min at 37°C of incubation, trypsinization was deactivated with 10 mL of DMEM F12 media. Then, 15 mL of the cells got transferred to a 15 mL falcon and were centrifuged at 200 g for 5 min. The liquid got removed, and 2 mL of DMEM F12 media was added and mixed. Afterward, the cells were diluted to 1–2 × 10^4^ cells/mL and added per well of a 48-well plate in a total volume of 500 µL per well. Before the cells were added, all samples were washed in sterile distilled water and sterilized with UV-light for 1 h at 254 nm with 40 μW/cm^2^ intensity (ESCO SC2-6E1, UV-30A Lamp). For osteoblast growth on the upper circular area of the titanium cylinders, the upper sample area was sealed off with a silicone O-ring to fit into a well of a 48-well plate. After the addition of the cells to the titanium samples, the setup got incubated for 72 h at 34°C at 95% humidity and 5% CO_2_ atmosphere. The media was exchanged after 48 h of incubation. Osteoblast adhesion was checked after 72 h of cell culture. After removing the used media, each well was washed with 250 µL of PBS, the PBS was removed, and 500 µL of TRYPLE was added. After 5 min at 37°C of incubation, trypsinization of the cells has been deactivated by adding 500 µL of DMEM F12 media. The cells got extracted, and of each well, 1 mL of cell-containing liquid was added to a 1.5 mL reaction tube. The tubes were centrifuged at 200 g for 5 min. The media were removed, and 1 mL of DMEM F12 media was added. In the next step, the cells were counted with Trypan blue solution in a Neubauer improved chamber. To evaluate the expression levels of genes responsible for biofilm formation and proliferation of *hFOB 1.19* on titanium samples, the primers in Table 3 were designed and used.

**TABLE 3:** Primer tables for biofilm-related genes and cell stress-related genes for *Staphylococcus aureus ATCC 29213* and proliferation genes of *hFOB 1.19.*

*S. aureus ATCC 29213* genes	Forward primer	*hFOB 1.19* genes	Forward primer
	Reverse primer		Reverse primer
IcaA	5′- CGC ACT CAA TCA AGG CAT TA -3′	Runx 2.1	5′- CAC CAT GTC AGC AAA ACT TCT T -3′
	5′- CCA GCA AGT GTC TGA CTT CG -3′		5′- TCA CGT CGC TCA TTT TGC -3′
IcaB	5′- CAC ATA CCC ACG ATT TGC AT -3′	Runx 2.2	5′- GTG CCT AGG CGC ATT TCA -3′
	5′- TCG GAG TGA CTG CTT TTT CC -3′		5′- GCT CTT CTT ACT GAG AGT GGA AGG -3′
IcaC	5′- CTT GGG TAT TTG CAC GCA TT -3′	Col1A1	5′- CAA GAG TGG TGA TCG TGG TG -3′
	5′- GCA ATA TCA TGC CGA CAC CT -3′		5′- GCC TGT CTC ACC CTT GTC A -3′
IcaD	5′- ACC CAA CGC TAA AAT CAT CG -3′	Col2A1	5′- AGA GGG CAA TAGC AGG TTC A -3′
	5′- GCG AAA ATG CCC ATA GTT TC -3′		5′- GCG TGA GGT CTT CTG TGA CC -3′
SarA	5′- AAG GAC AAT CAC ATC ACG AAG -3′	ALPL	5′- CCA TCC TGT ATG GCA ATG G -3′
	5′- GAA CGC TCT AAT TCA GCG G -3′		5′- CGC CTG GTA GTT GTT GTG AG -3′
SodA	5′- GTT TCA TCA CGA CAA ACA TCA C -3′	BMP7	5′- TCA GCG TTT ATC AGG TGC TC -3′
	5′- TGA CAT CCT CAT CGC TTC C −3′		5′- CCA GAG GGT ACG GCT GTC -3′
SigB	5′- AGA AGC AAT GGA AAT GGG AC -3′	GAPDH	5′- AGC CAC ATC GCT CAG ACA C -3′
	5′- CTT AAA CCG ATA CGC TCA CC -3′		5′- GCC CAA TAC GAC CAA ATC C -3′

Before the extraction of bacterial RNA, 24 h of biofilm formation with *S. aureus ATCC 29213* on titanium samples was performed. To extract RNA from osteoblasts, 72 h of osteoblast proliferation of *hFOB 1.19* cells on titanium samples was performed. For each material, six samples were used to extract RNA. RNA extraction, cDNA synthesis step, and RT-qPCR analysis were adapted from the work of Spiegel et al. (2022).

To characterize biofilm formation and osteoblast proliferation on the titanium samples, images were taken using a scanning electron microscope (SEM, JSM-6010LV, JEOL GmbH). After biofilm formation and proliferation of osteoblasts, the titanium samples got fixed overnight in 500 µL of 2.5% glutaraldehyde. After fixation, the samples got dehydrated in an ascending alcohol series 50%–70%–80%–99.9% of ethanol for 5 min for each stage. The titanium cylinders were then fixed onto aluminum pins with Leit-C gluing strips (Göcke, Plano GmbH). The samples and pins were sputtered with gold for 40 s (Agar Sputter Coater, Agar Scientific Ltd.).

## 3 Results and discussion

### 3.1 Technological aspects

**FIGURE 2:**
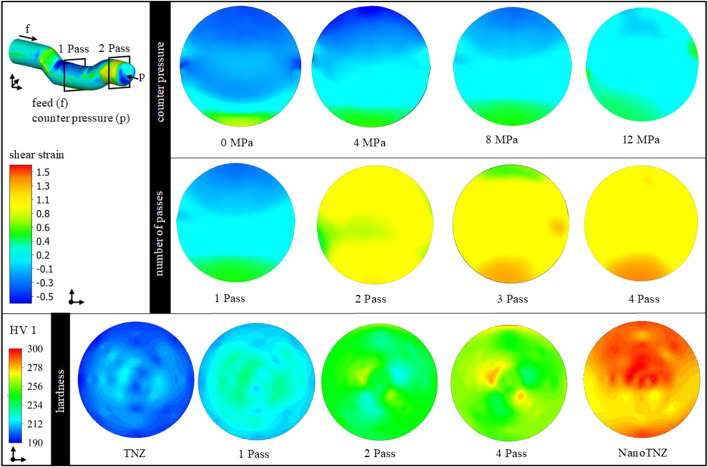
Contour plots of the shear strain distribution in cross-section as a function of the counter pressure and the number of passes. Bottom: contour plots of the hardness distribution in cross-section as a function of the material conditions and number of passes.

The influence of the counter pressure on strain distribution after the first pass is shown in Figure 2, first line. As can be seen, in case no counter pressure is applied, the strain distribution is inhomogeneous, which can be laid back to insufficient tool filling and superimposed bending. As the counter pressure increases, better tool filling is achieved. Increased homogeneity is obtained between a counter pressure of 4 and 8 MPa. In addition, a hydrostatic stress state is induced in the forming zone supporting damage-free forming, as already observed in previous studies for CP-titanium (Kluy et al., 2021). A counter pressure of 12 MPa leads to complete tool filling and the most homogeneous shear strain distribution. However, the simulation shows an inacceptable buckling of the bar in front of the ECAS tools. Hence, the maximum feasible counter pressure of 8 MPa has been used in experimental investigations to achieve a good balance between forming capability and homogeneity of the microstructure. Previous studies have shown that a rotation of the bar and the application of multiple passes (beyond two passes) do not significantly improve the microstructure (Klinge et al., 2022b), which seems to be confirmed by the simulation results presented here. The major advantages of two-pass ECAS (compared to a 1-Pass deformation) are increased homogeneity and straightness of the bars after processing as the first shearing procedure is followed by inverse shearing (Kluy et al., 2021). Hence, optimal ECAS processing is achieved by two-pass ECAS with a counter pressure of 8 MPa.

To validate the TNZ–ECAS process, the process limits were analyzed with respect to formability (e.g., crack and/or shear band formation). In addition, hardness distribution (contour plots) has been used as shown in Figure 2. The αʺ martensitic starting structure shows a homogeneous hardness distribution of 205 HV1 in the cross-section. After one-pass ECAS, the hardness increases to 230 HV1 due to strain hardening. However, the hardness distribution is inhomogeneous as expected from the aforementioned simulation results. After the second pass, the hardness further increases to 250 HV1 and is distributed homogeneously, which is in good correlation with the simulation results. Furthermore, forming of up to four passes shows a slight increase in hardness, whereas no significant improvement in homogeneity is achieved. This may indicate a saturation point in grain refinement.

**FIGURE 3:**
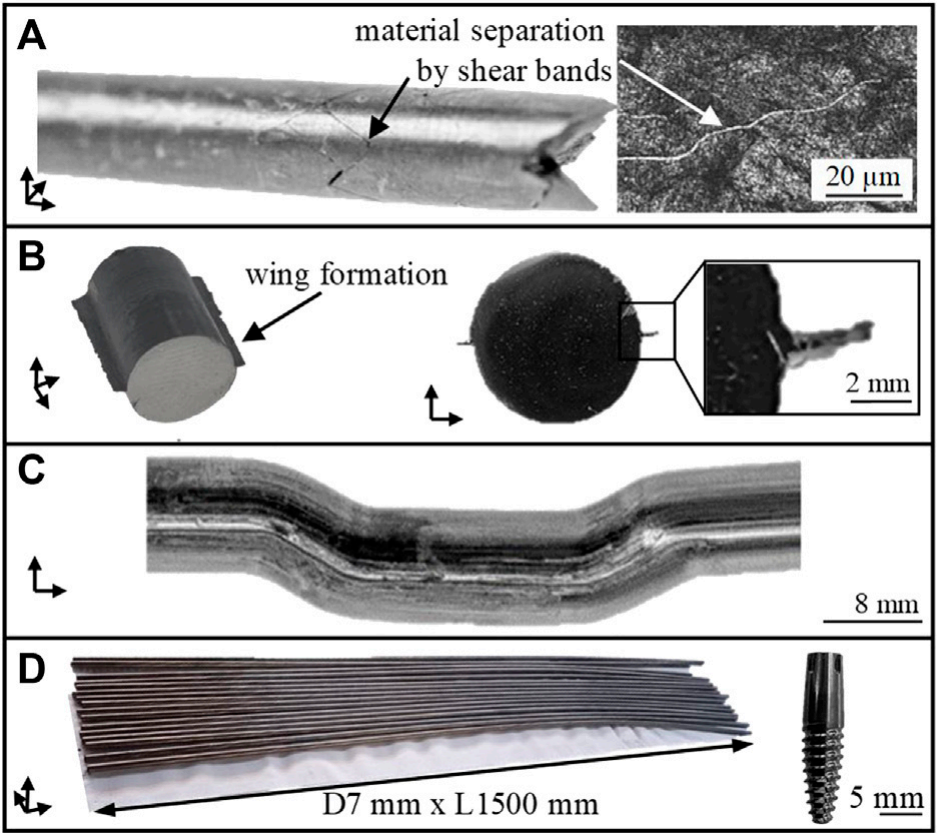
Process limits of TNZ in the ECAS process. Failure due to shear band **(A)**, wing formation **(B)**, successful forming **(C)**, and production of considerably long bars for dental implants **(D)**.

The process limits are shown in Figure 3. In case the as-received TNZ alloy is processed at low temperatures, the material fails due to shear band formation (A). Increasing forming temperatures reduce the flow stresses of TNZ and thus improve the forming capacity of the material. However, grain growth may occur at higher forming temperatures. In addition, a hydrostatic compressive stress state is generated in the forming zone by the counter pressure applied. However, increasing counter pressures to values exceeding 12 MPa results in material flow into the tool gap, the so-called wing formation (B). Damage-free forming (C) is only possible if 1) a soft martensitic initial state is used for ECAS deformation at a hydrostatic compressive stress state (i.e., a counter pressure of 8 MPa) and 2) a forming temperature of approximately 150°C. The production of considerably long bars (D) suitable for implant applications requires rotary swaging (for straightening) and subsequent heat treatments (Section 3.2) after two-pass ECAS. In this final material state, a homogeneous hardness distribution of 275 HV1 is determined. The process route has been reproduced several times to ensure process stability and allow the production of a larger amount of material.

### 3.2 Material scientific aspects

**FIGURE 4:**
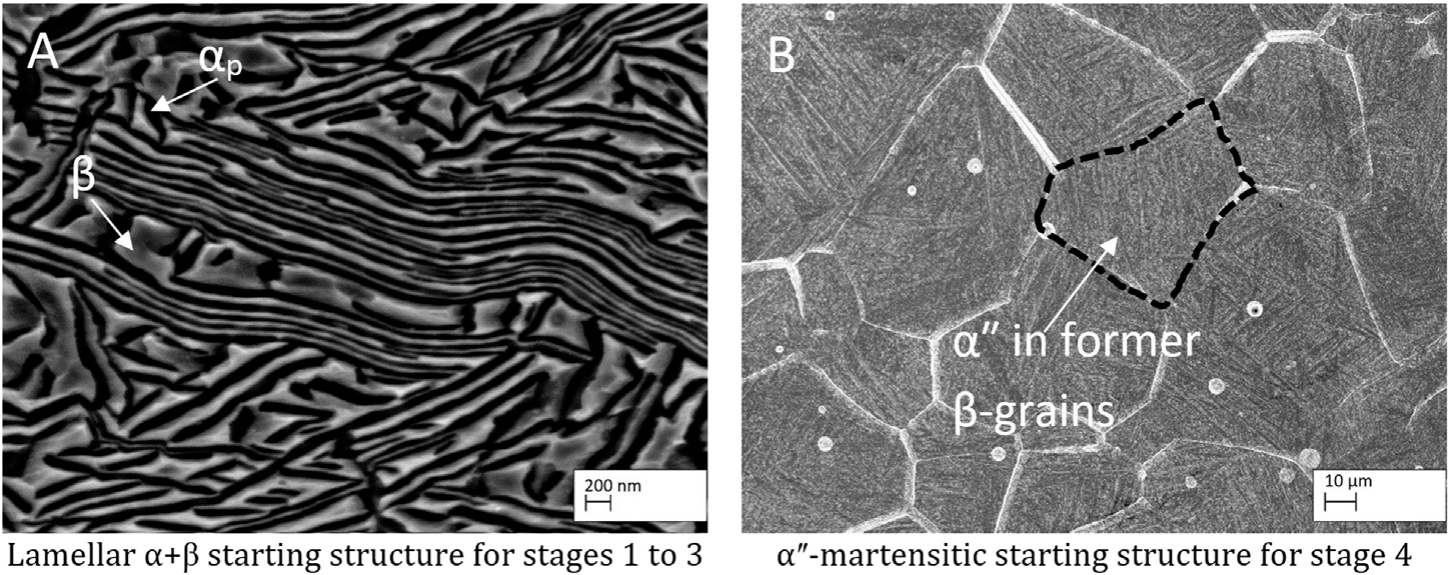
Starting microstructures for the different forming stages. **(A)** lamellar α+β-microstructure and **(B)** αʺ-martensitic microstructure.

Figure 4 shows the starting microstructures used for the different stages, namely, the starting structure for the stages 1–3 (A). It consists of deformed α_P_ (dark) and β (bright) lamellae after the radial forging to diameter 10 mm. The αʺ-martensitic starting structure used for stage 4 is shown in (B) and is composed of fine martensitic αʺ-needles within the former β-grains. EDS measurements show a homogeneous element distribution, as expected.

**FIGURE 5:**
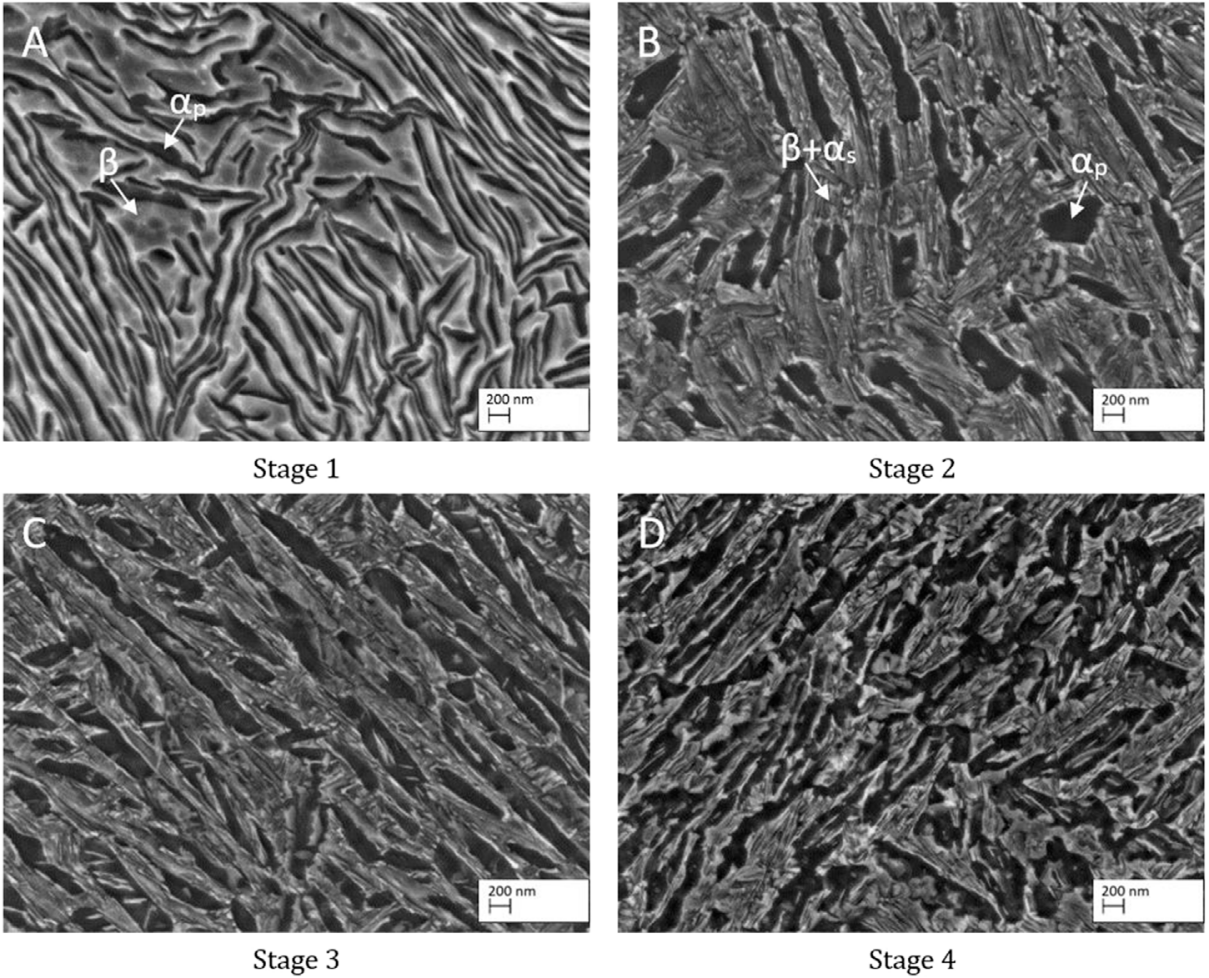
Microstructure of the four different TNZ stages: **(A)** stage 1, **(B)** stage 2, **(C)** stage 3, and **(D)** stage 4.

The related microstructures of the four different TNZ stages are shown in Figure 5. As EDS measurements on structures smaller than 2 µm are not feasible, XRD has been performed in previous studies (Klinge et al., 2021), confirming that, after rotary swaging (stage 1), the microstructure consists of highly deformed α and β lamellae. Small amounts of (stress induced) αʺ-martensite might be present as well but have not been detected by XRD. This can be explained by 1) a potential low volume fraction less than 3%, 2) a relatively poor signal-to-noise ratio in the XRD experiments, and 3) the fact that the three main peaks of the αʺ-phase superimpose with the {101} α-reflection. After the additional heat treatment (stage 2), the microstructure shows a mixture of globular and lamellar-like α_P_-grains, indicating that the recrystallization did not lead to a complete globularization of the α-phase. This could either be a result of the relatively short recrystallization time and/or an indication of insufficient shearing. After recrystallization at 700°C, the microstructure consists of α_P_ grains in an αʺ matrix after water quenching (not shown here). Subsequent ageing leads to (partial) αʺ decomposition into small α_S_ and β grains, leading to the nanostructured microstructure. The microstructure of stages 3 and 4 is similar to that of stage 2. Due to the additional ECAS step for stage 3, the microstructure is finer than that observed at stage 2. The finest microstructure is reached at stage 4, which can be explained by the fine αʺ-starting structure. After the first rotary swaging, the subsequent ECAS process, and the final rotary swaging, the martensite needles are highly sheared, providing a comparably large number of recrystallization seeds. The recrystallization heat treatment then leads to an UFG microstructure as intended, which can be further refined (nanostructured) by the ageing treatment.

**FIGURE 6:**
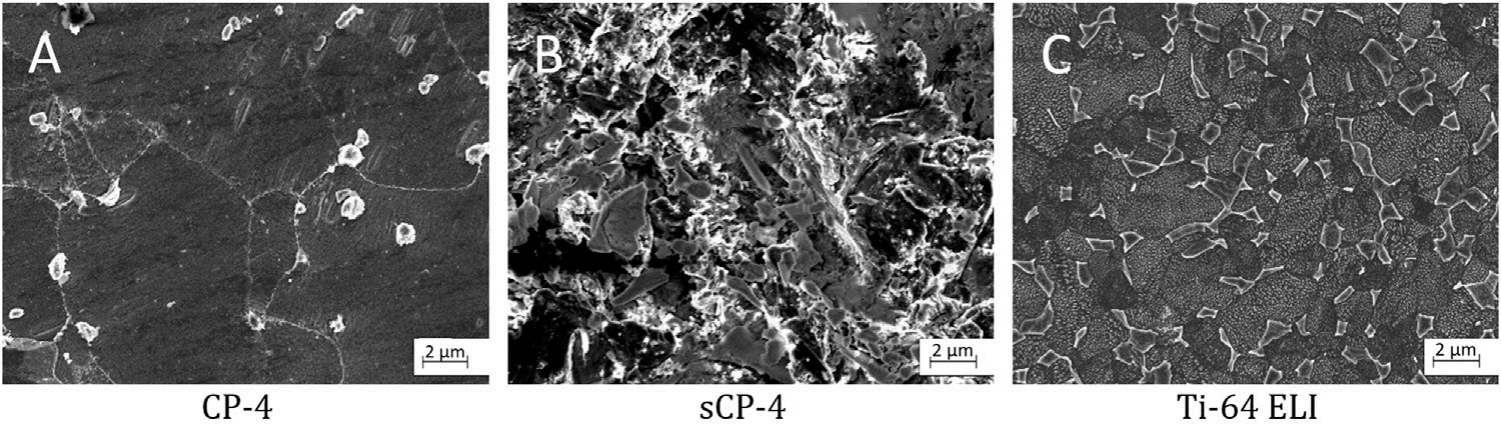
Microstructures of the reference materials: **(A)** CP-Titanium Grade 4, **(B)** sandblasted CP-Titanium Grade 4, and **(C)** Ti-6Al-4V ELI.

Figure 6 shows the microstructure of CP-4 in the AR (A) and sandblasted (B) condition, as well as the AR condition of Ti-64 ELI (C). The white particles visible in (A) have been identified as preparation artefacts as CP-titanium is comparably soft so that small particles can be pressed into the surface during sample preparation. Nevertheless, the grain structure is clearly visible showing a mixture of small and coarse globular *α*_p_ grains. The sandblasted surface is comparably rough with dents and structures of all shapes and sizes. The Ti-64 ELI microstructure consists of globular *α*_p_ grains surrounded by the β-phase.

**FIGURE 7:**
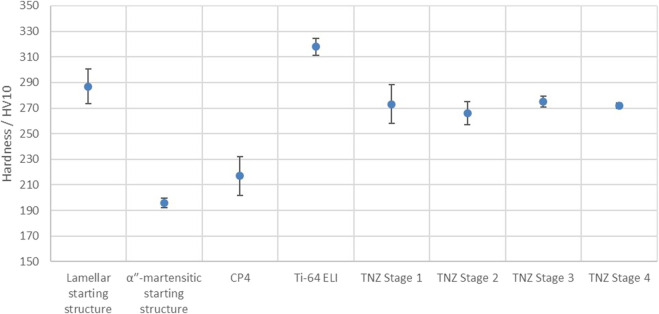
Hardness values of the investigated materials.

The hardness values of all the investigated materials (CP-4, Ti-64 ELI, and TNZ at the four different stages), as well as those of the two TNZ starting structures used for the ECAS process, are shown in Figure 7. As can be seen, the hardness of the lamellar starting structure is comparably high (287 HV10) due to work hardening so that the formation of adiabatic shear bands during the rotary swaging can be understood. During the additional heating step, work hardening is reduced, and after water quenching, the soft αʺ-martensite develops (Brunke, 2019), leading to a hardness of 196 HV10. In addition, after pre-heating, the hardness distribution is more homogeneous compared to the radially forged stage. This can be explained by annealing the alloy above β_T_ so that the thermo-mechanical history of the specimen is removed. As expected, the hardness of CP-4 (217 HV10) is much lower than that of Ti-64 ELI (318 HV10). The hardness of the different TNZ stages 1–4 is similar, 273, 266, 275, and 272 HV10, respectively, whereas the deviation of stages 1 and 2 is larger than that of stages 3 and 4. This once again supports the observation (Section 3.1) that the ECAS process contributes to a more homogenous microstructure.

Table 4 shows the results of the tensile tests of the final process stage (stage 4) together with the requirements of ASTM F1713 and the goals of this study. In addition, the mechanical properties of the two starting structures and the standard materials, CP-4 and Ti-64 ELI, are presented. Young’s modulus of the lamellar-starting structure measured in the hysteresis loop is very low (28 GPa), indicating that, besides α and β lamellae, also a small amount of (stress-induced) αʺ-martensite should have been formed during radial forging. Young’s modulus measured in the first elastic straight line was 64 GPa, which is comparably low as well. The work hardening after radial forging results in a very high UTS of 1,039 MPa at a very low elongation at fracture of 1.7%. The fully αʺ-martensitic-starting structure, on the other hand, has low Young’s modulus of 51 GPa, a moderate UTS of 632 MPa, and a large elongation at fracture of 23.7%.

**TABLE 4:** Mechanical properties of the final forming stage (stage 4), as well as the requirements of the medical standard ASTM F1713 and internal set goals together with the mechanical properties of the starting microstructures.

	*E/*GPa	*E*_*Hysteresis*_/GPa	*UTS*/MPa	*A*/%
ASTM F1713	n. def	n. def	860	8.0
Goals of this study	70–80	70–80	950	8.0
Lamellar starting structure	64	28	1,039	1.7
αʺ-Martensitic starting structure	58	51	632	23.7
CP-4	95	96	650	13.4
Ti-64 ELI	109	102	1,086	12.2
TNZ stage 4	83	73	994	9.6

CP-4 and Ti-64 ELI both have high Young’s modulus of 95 GPa and 109 GPa (96 GPa and 102 GPa for the hysteresis loop), respectively. The elongation at fracture of 13.4% for Ti-64 ELI is larger than the 10% required in the related specification. However, the elongation of 12.2% measured for CP-4 is lower than the required 15%. However, the test certificate of the material supplier states an elongation at fracture of more than 15%. As our CP-4 tensile test specimens failed off-center, the elongation at fracture might have been underestimated. The UTS for CP-4 was 650 MPa and the UTS of Ti-64 ELI is significantly higher (1,086 MPa) as expected.

As mentioned previously, for the production of stages 1–3, the lamellar microstructure has been used so that shear band formation occurred during the rotary swaging. If a shear band is present in a tensile test specimen, premature failure can be expected so that no tensile properties can be provided here. The most promising mechanical properties are obtained for stage 4 samples, namely, Young’s modulus of 83 GPa (73 GPa measured in the hysteresis loop) and an UTS of 994 MPa at an elongation at fracture of 9.6% (fulfilling the requirements specified in related mechanical standard ASTM F1713 and the project goals).

### 3.3 Biological aspects

**FIGURE 8:**
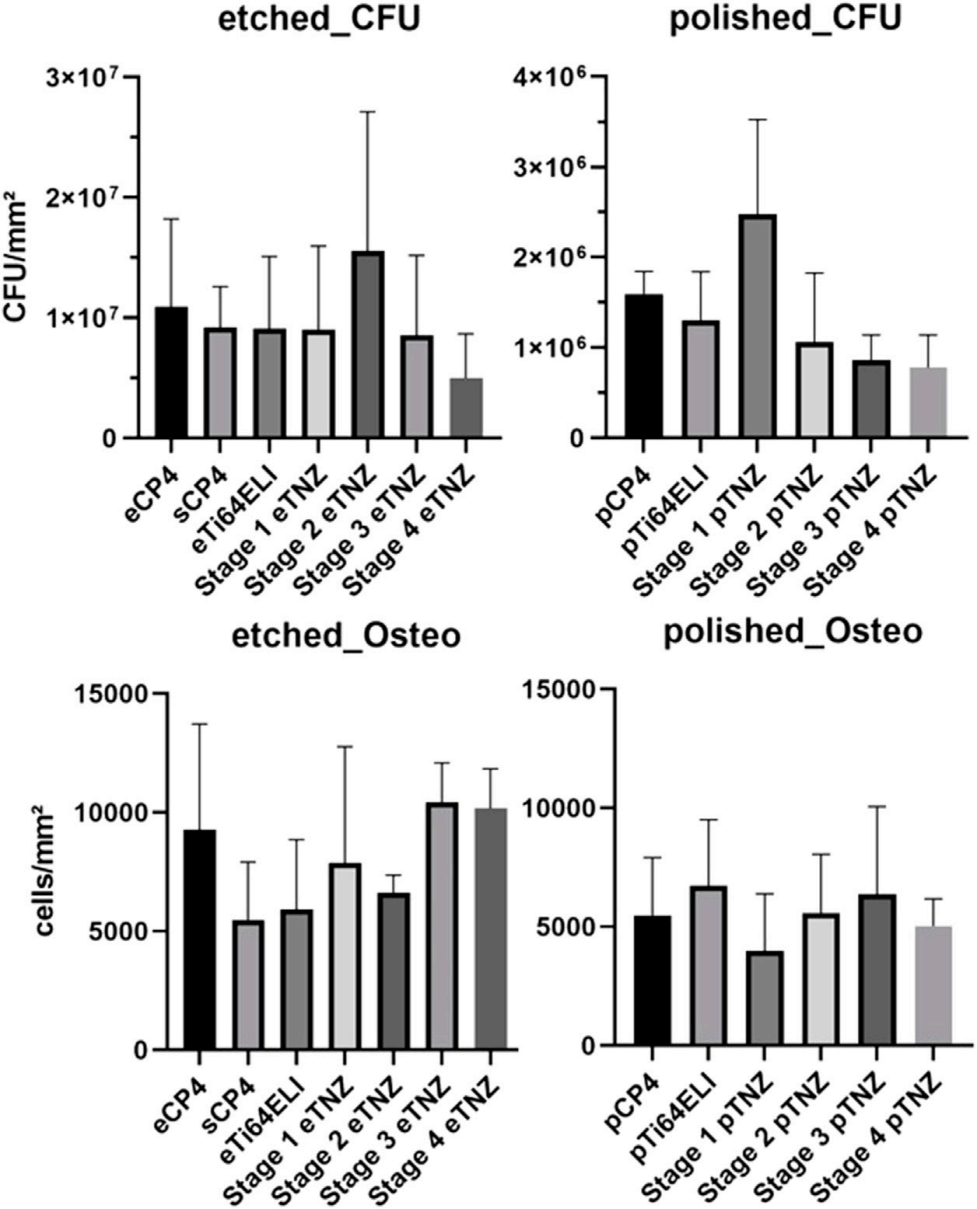
Bacterial biofilm formation and osteoblast proliferation cell counts per area on etched and polished surfaces.

Colony forming units on all etched titanium alloys investigated in our study showed no significant differences (Figure 8). The lowest count of CFU on etched material occurred on stage 4 material with 5.01 × 10^6^ CFU/mm^2^ (SD: ± 3.56 × 10^6^ CFU/mm^2^), whereas the highest CFU count was found on etched stage 2 material with 15.56 × 10^7^ CFU/mm^2^ (SD: ± 1.15 × 10^7^ CFU/mm^2^). The two commercially available titanium alloys, CP-4 and Ti-64 ELI, showed CFU counts of 1.09 × 10^7^ CFU/mm^2^ (SD: ± 7.3 × 10^6^ CFU/mm^2^) and 9.09 × 10^6^ CFU/mm^2^ (SD: ± 6.0 × 10^6^ CFU/mm^2^), respectively. However, sCP-4 material had CFU counts of 9.19 × 10^6^ CFU/mm^2^ (SD: ± 3.38 × 10^6^ CFU/mm^2^), while stage 1 and stage 3 materials had CFU counts of 9.0 × 10^6^ CFU/mm^2^ (SD: ± 6.96 × 10^6^ CFU/mm^2^) and 8.54 × 10^6^ CFU/mm^2^ (SD: ± 3.65 × 10^6^ CFU/mm^2^), respectively.

On polished samples, significant differences in the CFU count between stage 3 and stage 4 TNZ as well as for CP-4 material can be observed. Stage 4 and stage 3 samples had the lowest CFU count with 7.79 × 10^5^ CFU/mm^2^ (SD: ± 3.35 × 10^5^ CFU/mm^2^) and 8.58 × 10^5^ CFU/mm^2^ (SD: ± 2.78 × 10^5^ CFU/mm^2^), respectively. The highest CFU count occurred on stage 1 samples with 2.47 × 10^6^ CFU/mm^2^ (SD: ± 1.04 × 10^6^ CFU/mm^2^). The commercially used materials, CP-4 and Ti-64 ELI, had CFU of 1.59 × 10^6^ CFU/mm^2^ (SD: ± 2.5 × 10^5^ CFU/mm^2^) and 1.29 × 10^6^ CFU/mm^2^ (SD: ± 5.4 × 10^5^ CFU/mm^2^), respectively. Stage 2 material showed 1.06 × 10^6^ CFU/mm^2^ (SD: ± 7.64 × 10^5^ CFU/mm^2^).

Osteoblast proliferation and adhesion was the highest on etched stage 3 samples (10,433 cells/mm^2^) and the second highest on stage 4 samples (10,168 cells/mm^2^), as shown in Figure 8. On CP-4 samples, osteoblasts grew with 9,284 cells/mm^2^ and on Ti-64 ELI with 5,924 cells/mm^2^. On polished samples, osteoblast cell count deviated with no significant differences, while stage 1 showed the highest count with 6,713 cells/mm^2^ and stage 4 showed the lowest count with 3,978 cells/mm^2^.

**FIGURE 9:**
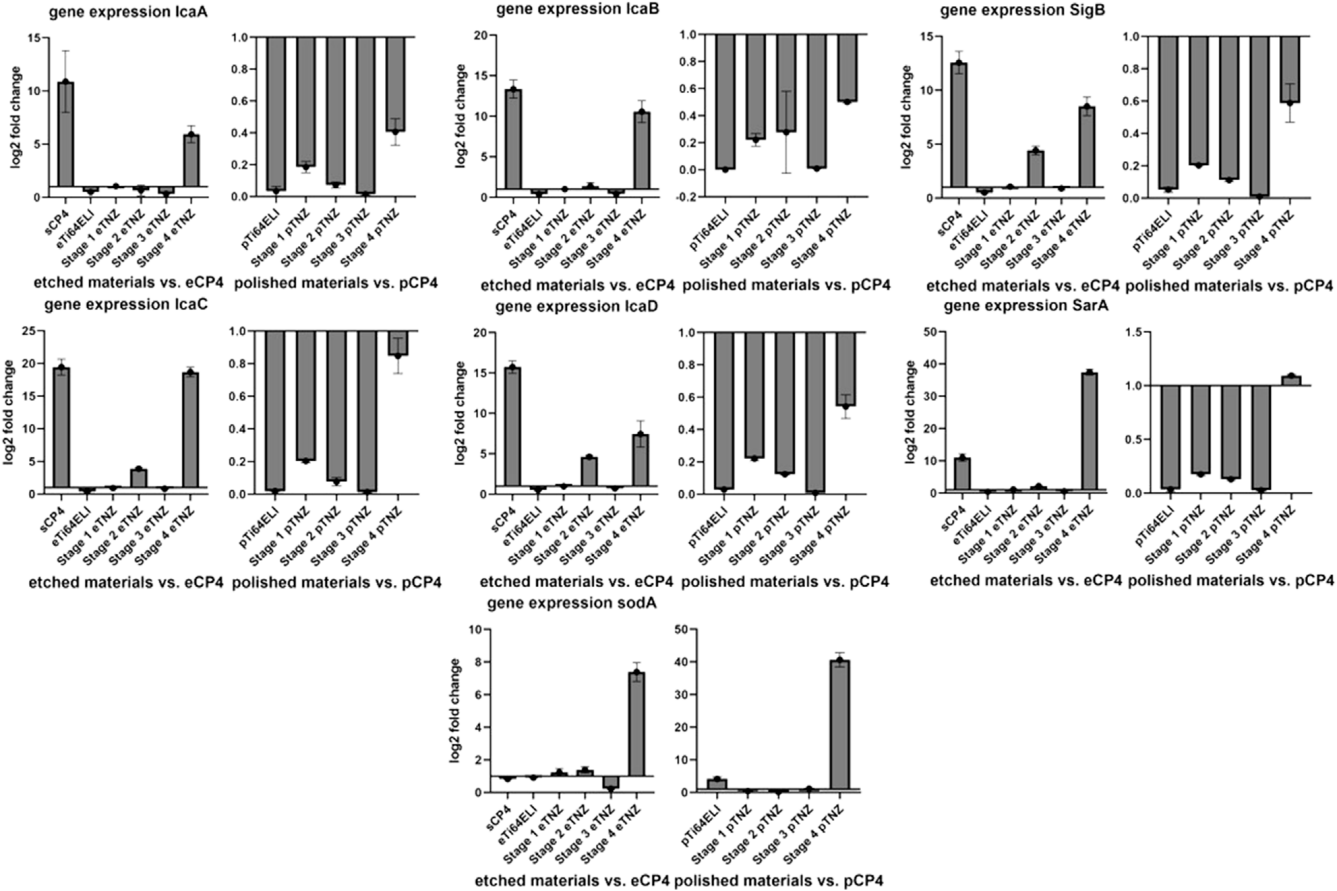
Expression of biofilm formation-related genes and cellular stress-related genes in *Staphylococcus aureus ATCC 29213* on etched, polished, and sandblasted surfaces. IcaABCD are regulating the expression of polysaccharide intercellular integrin. SigB is responding to cellular stress and regulating SarA, which is regulating IcaADBC and sodA. SodA is regulating the expression of superoxide dismutase A, a reactive oxygen species binding protein. All gene expression fold changes are compared relatively to the gene expression on polished or etched CP-4 material.

The bacterial biofilm gene expression of IcaA showed a higher expression on etched sCP-4 and etched stage 4 samples with 10.898 and 5.932 higher folds in gene expression (Figure 9). IcaB also showed a higher gene expression on sCP-4 and etched stage 4 samples of 13.346 and 10.567 folds, respectively. On both materials, sCP-4 and etched stage 4, the gene expression of IcaC and IcaD was higher than on etched CP-4. IcaC showed 19.447 folds higher gene expression on sCP-4 and 18.686 folds higher gene expression on etched stage 4 samples, whereas IcaD showed 15.735 on sCP-4 and 7.448 folds higher gene expression on etched stage 4 samples. However, IcaD was also higher in expression on stage 3 TNZ with 4.629 higher gene expression in comparison to etched CP-4. On polished samples, no higher gene expression was observed in the Ica-cascade. The gene expression of SigB and SarA were both higher on sCP-4, etched stage 4, and etched stage 3 TNZ. The SarA gene expression was also higher expressed on polished stage 4 samples. The sodA gene was higher expressed on etched stage 4 samples, with 7.385 folds higher gene expression, whereas on polished stage 4 samples, the gene expression was also higher with 40.627 higher folds of gene expression. On polished Ti-64 ELI samples, the gene expression of sodA was raised by 4.154 folds.

The upregulation of biofilm-related genes (IcaA, D, B, and C) and cell stress-related genes (SigB, SarA, and sodA) indicate an antibacterial influence on *S. aureus ATCC 29213* due to cell stress (Chan et al., 1998; Ballal and Manna, 2009; Morrison et al., 2012). As SigB and SarA are both involved in cell stress and biofilm upregulation, a higher regulation of both genes is to be expected when Ica-cascade genes and superoxide dismutase gene soda are also upregulated.

**FIGURE 10:**
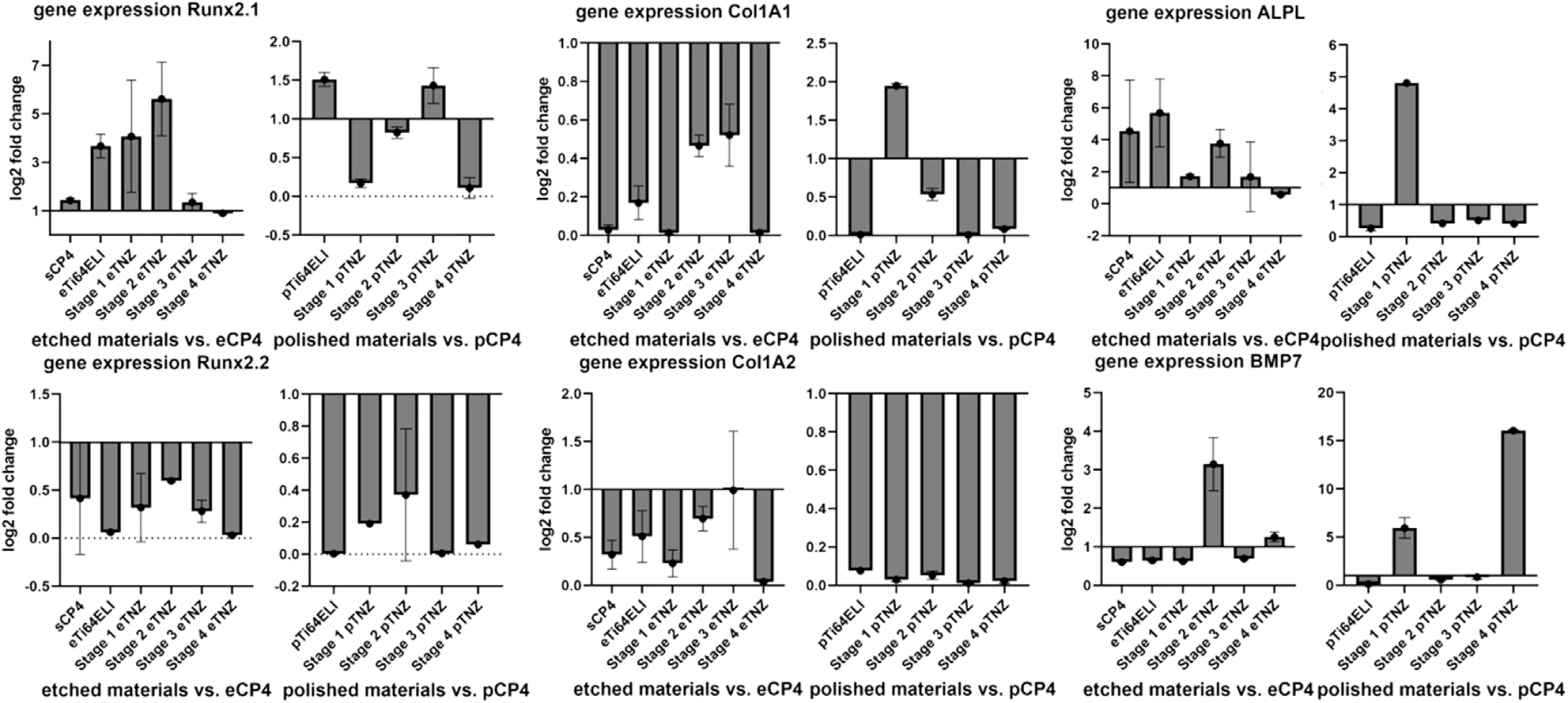
Expression of proliferation-related genes of *hFOB 1.19* on etched, polished, and sandblasted surfaces. RunX 2.1 and 2.2 are both regulating proliferation of osteoblasts. Col1A1 and Col1A2 are both involved in the regulation of collagene expression. ALPL regulates the expression of alkaline phosphatase, which is more expressed during proliferation processes. BMP7 is involved in inhibiting the proliferation processes and initiating cell differentiation. All gene expression fold changes are compared relatively to the gene expression on polished or etched CP-4 material.

The proliferation gene expression of *hFOB 1.19* cells showed an upregulation on etched stage 2 (5.619-fold), etched stage 1 (4.072-fold), and etched Ti-64 ELI (3.676-fold) in comparison to etched CP-4 within the RunX 2.1 gene (Figure 10). The RunX 2.1 gene expression was slightly upregulated on Ti-64 ELI (1.510-fold) and stage 3 samples (1.432-fold) when the surface has been polished. Alkaline phosphatase gene expression was upregulated on etched Ti-64 ELI (5.689-fold), sCP-4 (4.541-fold), etched stage 2 (3.777-fold), etched stage 1 (1.712-fold), and etched stage 3 samples (1.681-fold). The gene expressions of ALPL and on Col1A1 were both increased on polished stage 1 samples (4.812- and 1.948-fold). The proliferation inhibiting BMP7 gene expression was upregulated on etched stage 2 samples with 3.147-fold higher expression and on etched stage 4 samples with 1.258-fold higher expression. On polished surfaces, BMP7 gene expression was upregulated on stage 4 samples (16.056-fold) and on stage 1 samples (5.947-fold). The downregulation of BMP7 indicates cell proliferation as BMP7 is inhibiting osteoblast proliferation and stimulates cell differentiation (Setzer et al., 2009).

**FIGURE 11:**
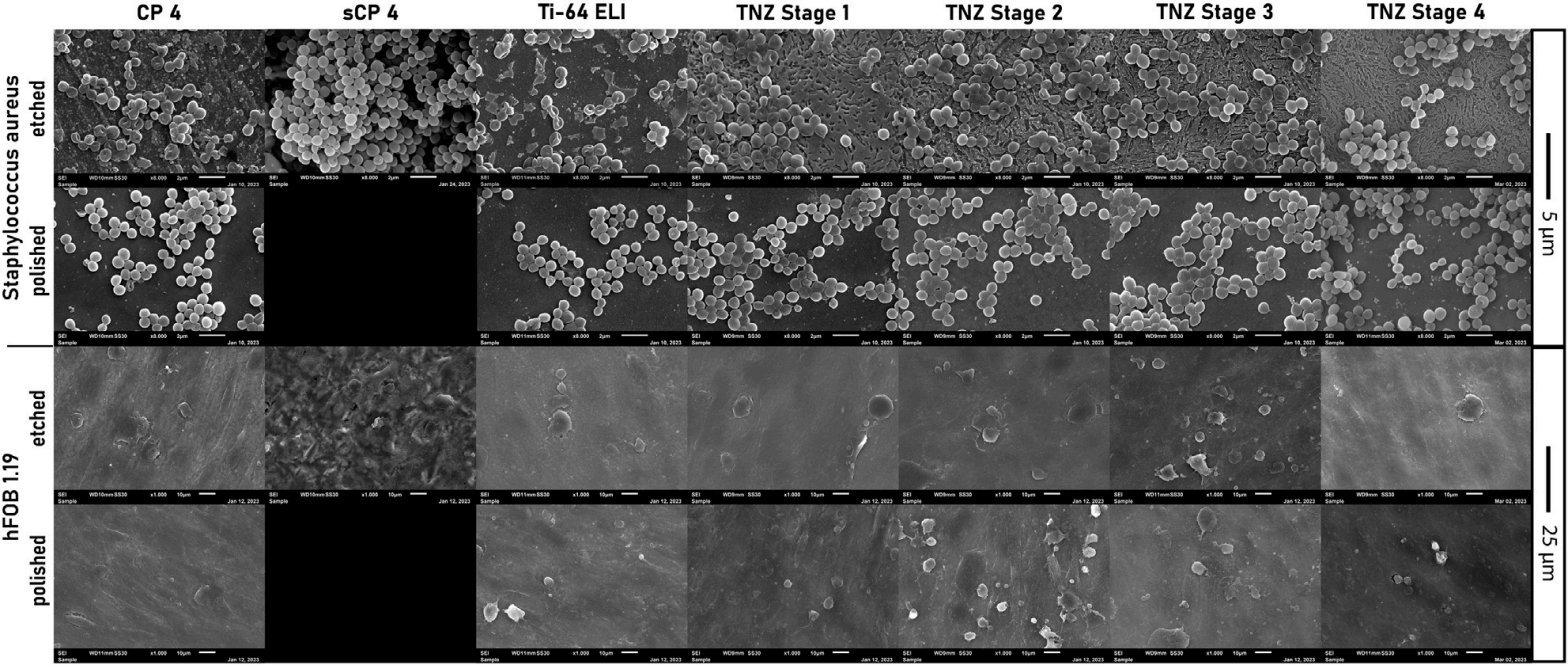
Images of scanning electron microscopy of *Staphylococcus aureus ATCC 29213* and *hFOB 1.19* on all used etched, polished, and sandblasted materials.

The bacterial cell morphology of *S. aureus ATCC 29213* on etched stage 4 differed compared to the cell morphology on CP-4 and Ti-64 ELI (Figure 11). The cells on CP-4, Ti-64 ELI, and stages 1 and 2 are more roundly shaped, sometimes with a hole in the middle of the cells. On etched stage 4 samples, the bacteria seem to be more fractured and roughed. Cells with a hole in the middle are less common. The more fractured shape indicates cellular stress. This morphological observation may correspond to the observed lower CFU count and higher gene expression of cell stress genes. Scanning electron microscopy images showed confluent osteoblasts on all examined materials.

The examined etched stage 4 material showed lower bacterial growth after 24 h of incubation and higher genes expression of biofilm genes and cells stress genes. In addition, SEM analyses showed more fractioned cell morphology. Aguilera-Correa et al. (2017) connected the antibacterial properties to the production of hydroxyl radicals on the TNZ alloy. As the formation of hydroxyl radicals is resulting in oxidizing stress for the bacterial cells, our findings of increased expression of superoxide dismutase A (sodA) are matching to the literature. It has been reported that nanoparticles are increasing the reactive oxygen species (ROS) formation (Attarilar et al., 2020). Therefore, the identification of an antibacterial effect on stage 4 samples, whose nanostructure is increased by the ECAS process and subsequently RS, is matching with literature findings. However, etched and polished stage 4 samples had higher levels of expression in SigB, SarA, and SodA, indicating increased bacterial cell stress, probably caused by ROS. These findings translate to a lower CFU in both surface modifications, etched and polished, as well. Etched stage 4 and etched CP-4 allowed higher osteoblast proliferation after 72 h of incubation, leading to already reduced cell proliferation gene activity, higher cell counts, and more length of cell shape. Higher BMP7 expression indicates a completed cell proliferation and an already initiated cell differentiation of *hFOB 1.19* (Nemcakova et al., 2022). Etched stage 4 TNZ alloy seemed to be as biocompatible as etched CP-4. In previous studies of Park et al. (2011), osteoblasts showed increased proliferation on TNZ alloys. As surface modifications are crucial for osteoblast adhesion and proliferation, nanostructured surfaces are stimulating osteoblasts to proliferate due to increased attachment to integrins (Lopes et al., 2019). In comparison of the cell count of osteoblasts between etched Ti-64 ELI to the other titanium alloys, the lower osteoblast count can be related to the vanadium content of the alloy. It has been reported that vanadium has cytotoxic activity (Niinomi et al., 2016). On polished surfaces, the stage 4 and stage 3 materials showed the lowest amount of biofilm formation but no differences in osteoblast adhesion in comparison to other polished surfaces. Furthermore, the increased osteoblast count on etched stage 4 materials can be explained, considering the already stopped proliferation gene activity and swiftly increased BMP7 activity, to an already reached confluency in the experimental setup. Compared to all materials and surface modifications, etched stage 4 samples showed the highest osteoblast proliferation and the most pronounced reduction of bacterial cells.

## 4 Conclusion, summary, and outlook

In this study, coordinated research activities (production engineering, materials science, and microbiology) on the TNZ alloy have been conducted in a multidisciplinary approach at TU Darmstadt, TU Braunschweig, and the Medical University of Innsbruck. In particular, a combination of severe plastic deformation (i.e., ECAS) and subsequent recrystallization and ageing treatments were applied to achieve nanostructured microstructures to minimize bacterial colonization and promote osteoblast growth. The biological tests were performed at different forming stages and heat treatment conditions of the TNZ alloy and were compared to standard biomedical alloys CP-Titanium Grade 4 and Ti-6Al-4V ELI (“as-received” and sandblasted condition).

As a result of the technological studies, the ECAS production of nanostructured TNZ as long and industrially usable bars has been enabled, and related processing conditions have been identified. To homogenize the microstructure and achieve damage-free forming, the influence of counter pressure and multiple passes on shear strain and hardness distribution was investigated. Process limits arise due to the shear band and wing formation. Damage-free homogeneous forming is possible by using a fully (soft) αʺ-martensitic starting structure, applying a hydrostatic compressive stress state with a counter pressure of 8 MPa, two-pass ECAS, and an elevated forming temperature.

The recrystallization and ageing (700°C/10 min WQ + 500°C/1 h AC) of ECAS produced bars leading to nanostructured microstructures with substructures smaller than 200 nm due to (partial) αʺ-martensite decomposition into the smaller α_s_+β-phase. In addition, the ECAS process leads to a more homogenous hardness and microstructure distribution compared to radially forged (“conventionally processed”) TNZ bars.

The biological tests showed that stage 4 TNZ (two-pass ECAS + rotary swaged + recrystallized and aged) has the most promising potential in reducing bacterial biofilm formation and increasing cell proliferation of osteoblasts compared to the standard biomedical alloys CP-4 and Ti-64 ELI. However, an etched surface had a larger impact on osteoblast proliferation than a polished surface.

Future work will now concentrate on the production of ECAS-processed TNZ dental implant demonstrators, including fatigue tests and an upscaling of the material and implant production to industrial scale. Afterward, clinical studies at the final product are planned to allow the certification process as a last step. Another interesting aspect to be investigated in future projects is the effect of the wettability on the biological properties.

## Data Availability

The original contributions presented in the study are included in the article/Supplementary Material. Further inquiries can be directed to the corresponding authors.
